# Effect of in-hospital delay on acute appendicitis severity: does time really matter?

**DOI:** 10.1007/s13304-024-01823-5

**Published:** 2024-04-02

**Authors:** Stefano Agnesi, Gabriele Mauro Di Lucca, Fabio Benedetti, Luca Fattori, Luca Degrate, Linda Roccamatisi, Marco Braga, Marco Ceresoli

**Affiliations:** Department of General and Emergency Surgery, School of Medicine and Surgery, University of Milano-Bicocca, 20900 Monza, Italy

**Keywords:** Acute appendicitis, Appendicectomy, Complicated appendicitis, Alvarado score, In-hospital surgical delay, Timing

## Abstract

Appendicitis is one of the most common abdominal emergencies. Evidence is controversial in determining if the in-hospital time delay to surgery can worsen the clinical presentation of appendicitis. This study aimed to clarify if in-hospital surgical delay significantly affected the proportion of complicated appendicitis in a large prospective cohort of patients treated with appendectomy for acute appendicitis. Patients were grouped into low, medium, and high preoperative risk for acute appendicitis based on the Alvarado scoring system. Appendicitis was defined as complicated in cases of perforation, abscess, or diffuse peritonitis. The primary outcome was correlation of in-hospital delay with the proportion of complicated appendicitis. The study includes 804 patients: 278 (30.4%) had complicated appendicitis and median time delay to surgery in low-, medium-, and high-risk group was 23.15 h (13.51–31.48), 18.47 h (10.44–29.42), and 13.04 (8.13–24.10) h, respectively. In-hospital delay was not associated with the severity of appendicitis or with the presence of postoperative complications. It appears reasonably safe to delay appendicectomy for acute appendicitis up to 24 h from hospital admission. Duration of symptoms was a predictor of complicated appendicitis and morbidity. Timing for appendicectomy in acute appendicitis should be calculated from symptoms onset rather than hospital presentation.

## Introduction

Acute appendicitis occurs at a rate of about 90–100 patients per 100,000 inhabitants per year in developed countries and it is one of the most common surgical emergencies worldwide, with an estimated lifetime risk reported to be 7–8% [1].

Traditionally, perforated appendicitis was considered to be the natural evolution of simple acute appendicitis, leading to a fast surgical approach. However, evidence is controversial in determining the consequences of delaying an appendicectomy for acute appendicitis [2–5].

A study by Alore et al. [3] showed that appendicectomies performed between 48–72 h from hospital admission had an increased 30-day mortality and higher rate of all major postoperative complications compared to those appendicectomies performed within 24 h or between 24–48 h from hospital admission. Moreover, Bolmers et al. [6] suggested that patients who underwent surgery for complicated appendicitis within 8 h experienced fewer postoperative complications and required fewer re-interventions than those patients who underwent surgery after the 8-h mark.

On the other hand, other studies argued that in-hospital delay in performing appendectomies in acute appendicitis is not associated with a poorer outcome. A retrospective study by Shin et al. [7] found no differences between patients with acute appendicitis operated within or after 8 h in terms of length of postoperative hospital stay, complication rate, and readmission rate. These results are consistent with those of Terrón-Arriaga et al. [8] who showed that an in-hospital delay of more than 8 h does not significantly increase the risk of complicated appendicitis, the occurrence of perioperative complications, the duration of postoperative hospitalization, or mortality. In addition, a study by Drake et al. [9] analyzed 9048 patients who underwent appendectomy across 52 hospitals in Washington State. They found no association between perforation and elapsed time from hospital presentation to surgery.

A recent meta-analysis by van Dijk et al. [2] including 45 studies with 152,314 patients, demonstrated that a delay of 24–48 h from hospital admission was not associated with a higher risk for complicated appendicitis of postoperative complications.

This study aimed to evaluate the effect of in-hospital delay on patients with clinical diagnosis of uncomplicated appendicitis treated with appendectomy.

## Materials and methods

This is a retrospective analysis of a prospectively collected register of all consecutive patients with acute appendicitis treated with appendicectomy at San Gerardo Hospital, Monza, between September 2013 and April 2021. For each patient were collected: age, sex, time from symptom onset; time between admission and surgery, length of surgical procedure and hospital stay, surgical approach, conversion rate, presence of surgical drains, postoperative complications.

Preoperative risk for acute appendicitis was evaluated with the Alvarado score and patients were grouped based on predicted risk: low risk (score 0–4), medium risk (score 5–6), and high risk (score 7–10) [10].

Patients with a preoperative clinical diagnosis of complicated appendicitis (diffuse peritonitis or radiological findings of perforation or abscess) were excluded from the analysis. Patients treated with appendectomy for a diagnosis different from acute appendicitis (tumor, endometriosis, presence of parasites (Enterobius vermicularis) in the appendicular lumen) and patients with negative appendectomy (no signs of inflammation at the pathological examination) were also excluded.

Patients were divided into two groups based on surgical findings: complicated or uncomplicated acute appendicitis. Acute appendicitis was considered as complicated in case of perforation, abscess, or diffused peritonitis.

The primary outcome of the study was the intraoperative diagnosis of complicated appendicitis. The secondary outcome was the development of postoperative complications.

Associations and correlations between study variables and outcomes were evaluated with univariate and multiple regression analysis. Categorical data were presented as percentages and were analyzed with the Chi-square test, while continuous variables were presented as median and interquartile range (IQR) and compared with the Mann–Whitney’s U test. Variables associated with the outcome at the univariate analysis were included in a multiple logistic regression model. All statistics were calculated using SPSS 26.0.

## Results

From September 2013 to April 2021, a total number of 1005 patients were operated for acute appendicitis. After the exclusion of 18 patients for histological findings that met exclusion criteria, 72 patients for negative appendectomy and 111 patients for a preoperative diagnosis of complicated acute appendicitis, a total of 804 patients were included in this study. Median age was 24 (14–46) years and 559 (61.1%) were men.

Three hundred eighty-one (41.6%) patients had a preoperative low risk of acute appendicitis (Alvarado 1–4), four hundred twenty-five (46.4%) medium risk (Alvarado 5–6), and one hundred nine (11.9%) high risk (Alvarado 7–10).

**Fig. 1:**
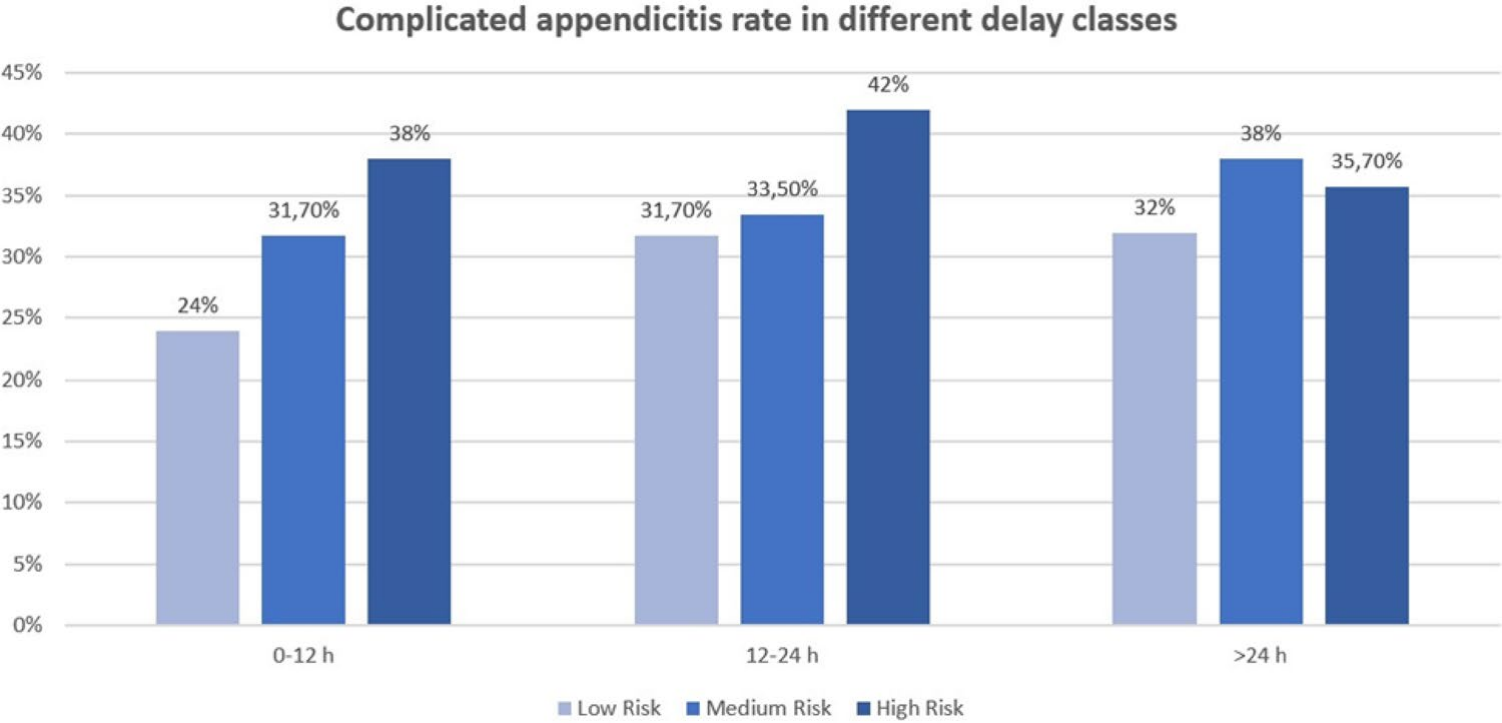
Complicated appendicitis rate in different delay classes

Median time from admission to surgery was 19.37 (11.19–29.90) h. Considering surgery delay for each risk class, low-risk group had a median time from admission to surgery of 23.15 (13.51- 31.48) h, while medium and high risk had, respectively, a median time to surgery of 18.47 (10.44–29.42) and 13.04 (8.13–24.10) h (see Fig. 1).

All patients were treated with appendectomy; median time of surgery was 70 min (IQR 55–90), 84% of them received laparoscopic appendectomy (769 patients) and the conversion rate was 9.1% (70 patients). Six hundred thirty-seven (69.6%) patients had an uncomplicated appendicitis while two hundred seventy-eight had a complicated one. Overall, morbidity was 10.7% and median comprehensive complication index of patients who experienced a postoperative complication was 20.9 (8.7–26.2).

Population details are summarized in Table 1.

**Table 1:** Whole population characteristics (915 patients)

Age (median, IQR)	24 (14–46)
Sex (male, %)	559 (61.1)
BMI (median, IQR)	21.80 (18.9–24.5)
History of previous abdominal surgery (yes, %)	73 (8)
Previous episodes (yes, %)	77 (8.4)
ABT (past 30 days) (yes, %)	31 (3.4)
Duration of symptoms (count, %)	
< 24 h	402 (43.9)
24–48 h	220 (24)
48–72 h	127 (13.9)
> 72 h	18.1 (166)
Fever (*T* > 37.5 °C) (yes, %)	188 (20.5)
Mc Burney (yes, %)	576 (62.2)
Blumberg (yes, %)	389 (42.6)
White blood cells (median, IQR)	14.49 (11.5–17.41)
Neutrophils (median, IQR)	11.73 (8.64 – 14.47)
CRP (median, IQR)	3.30 (0.64 – 8.84)
Alvarado score (median, IQR)	4 (2–6)
Alvarado risk class (count, %)	
Low risk	381 (41.6)
Medium risk	425 (46.4)
High risk	109 (11.9)
Complicated appendicitis	278 (30.4)
Type of surgery (laparoscopy, %)	769 (84)
Duration of surgery (min) (median, IQR)	70 (55–90)
Open conversion (yes, %)	70 (9.1)
Drainages (yes, %)	377 (41.2)
Complications (yes, %)	98 (10.7)
Surgical site infection (yes, %)	15 (1.6)
Abscess (yes, %)	27 (3)
Postoperative length of stay (days, median, IQR)	3 (2–5)
Clavien-Dindo (count, %)	
CD1	42 (42.9)
CD2	30 (30.6)
CD3a	6 (6.1)
CD3b	14 (14.3)
CD4a	4 (4.1)
CD4b	1 (1)
CD5	1 (1)
Comprehensive complication index (CCI) (median, IQR)	20.9 (8.7–26.2)

Table 2 shows the results of the univariate and multiple regression analysis of variables correlated with intraoperative findings of complicated acute appendicitis. Increasing age [OR: 1.024 (CI 95% 1.014–1.034) *p* < 0.001], duration of symptoms [24–48 h OR: 1.617 (CI 95% 0.997–2.623) *p* = 0.052; 48–72 h OR: 1.842 (CI 95% 1.041–3.259) *p* = 0.036; > 72 h OR: 2.079 (CI 95% 1.208–3.579) *p* = 0.008; neutrophils absolute count (OR: 1.058 (CI 95% 1.015–1.104) *p* = 0.008] correlated with the outcome.

**Table 2:** Population characteristic divided by appendicitis type—univariate and multivariate analysis (excluded patients with radiological evidence of complicated appendicitis)

	Uncomplicated appendicitis (633 patients)	Complicated appendicitis (171 patients)	Univariate analysis			Multivariate analysis		
			*p* Value	OR	95% CI	*p* Value	OR	95% CI
Age (median, IQR)	21 (13–37)	36 (15–50)	**< 0.001**	**1.024**	**1.015–1.032**	**< 0.001**	**1.024**	**1.014–1.034**
Sex (male, %)	388 (61.3)	113 (66.1)	0.252	0.813	0.570–1.159			
BMI (median, IQR)	21.05 (18.30–23.60)	22.45 (19.11–26.05)	0.106	1.055	0.989–1.126			
Previous abdominal surgery (yes, %)	41 (6.5)	17 (9.9)	0.123	1.594	0.881–2.883			
Previous episodes (yes, %)	59 (9.3)	10 (5.8)	0.154	0.604	0.302–1.208			
Antibiotics in the last 30 days (yes, %)	19 (3.0)	6 (3.5)	0.735	1.175	0.462–2.990			
Fever (> 37.5 °C) (yes, %)	115 (18.2)	45 (26.3)	**0.018**	**1.609**	**1.083–2.390**	0.075	1.491	0.961–2.314
Duration of symptoms								
< 24 h	306 (48.3)	56 (32.7)	**< 0.001**			**0.039**		
24–48 h	156 (24.6)	44 (25.7)	**0.054**	**1.541**	**0.993–2.392**	**0.052**	**1.617**	**0.997–2.623**
48–72 h	83 (13.1)	30 (17.5)	**0.008**	**1.975**	**1.191–3.274**	**0.036**	**1.842**	**1.041–3.259**
> 72 h	88 (13.9)	41 (24.0)	**< 0.001**	**2.546**	**1.595–4.063**	**0.008**	**2.079**	**1.208–3.579**
Mc Burney (yes, %)	381 (60.6)	110 (64.3)	0.371	1.174	0.826–1.668			
Blumberg (yes, %)	281 (44.5)	64 (37.4)	0.096	0.745	0.526–1.054			
WBC (median, IQR)	14.44 (11.30**–**17.17)	15.00 (12.36**–**17.96)	0.069	1.034	0.998–1.071			
Neutrophils (median, IQR)	11.52 (8.35**–**14.45)	12.52 (10.16**–**14.69)	**0.036**	**1.042**	**1.003–1.083**	**0.008**	**1.058**	**1.015–1.104**
C-reactive protein (median, IQR)	2.23 (0.51–6.15)	5.66 (1.59**–**13.40)	**< 0.001**	**1.042**	**1.022–1.063**	0.071	1.016	0.999**–**1.033
Alvarado risk class (count, %)								
Low risk	258 (40.8)	73 (42.7)	0.719					
Medium risk	306 (48.3)	77 (45.0)	0.524	0.889	0.620–1.275			
High risk	69 (10.9)	21 (12.3)	0.796	1.076	0.619–1.870			
Surgery delay (median, IQR)	19.48 (11.14–29.45)	19.15 (12.08–31.19)	0.741	0.998	0.987–1.009			
Surgery delay categorical (count, %)								
< 12 h	173 (27.3)	41 (24.0)	0.527					
12–24 h	214 (33.8)	65 (38.0)	0.268	1.282	0.826–1.989			
> 24	246 (38.9)	65(38.0)	0.625	1.115	0.720–1.725			

Statistical significance (*p* < 0.05) is highlighted in bold

No statistical association has been found between in-hospital surgery delay (expressed both as a continuous variable and as a categorical variable) and the severity of appendicitis. Even upon stratifying the population into Alvarado risk class groups, our analysis consistently demonstrates that the time to surgery remained unassociated with the severity of appendicitis across all risk classes. In particular, timing considered as a continuous variable was not associated with the severity of appendicitis in the low-, medium-, and high-risk classes, with *p* values of 0.86, 0.8, and 0.78, respectively. Moreover, when considering time as a categorical variable, for the low-risk group, the OR for surgery within 12–24 h was 0.64 (95% CI: 0.3–1.39), and for > 24 h, it was 1.23 (95% CI: 0.7–2.18). In the medium-risk group, the OR for surgery within 12–24 h was 0.95 (95% CI: 0.49–1.81), and for > 24 h, it was 1.14 (95% CI: 0.6–2). For the high-risk group, the OR for surgery within 12–24 h was 1.35 (95% CI: 0.41–4.48), and for > 24 h, it was 1 (95% CI: 0.25–4.1).

Predictors of postoperative morbidity were tested in the whole population. Table 3 shows the results.

**Table 3:** Predictive factor of postoperative complications (univariate and multivariate analysis)

	No postoperative complications (817 patients)	Postoperative complications (98 patients)	Univariate analysis			Multivariate analysis		
			*p* Value	OR	95% CI	*p* Value	OR	95% CI
Age (years)	**24 (14–44)**	**34 (17–58)**	**< 0.001**	**1.019**	**1.009–1.029**	0.121	1.010	0.997–1.023
Sex (male, %)	**490 (59.9)**	**69 (70.4)**	**0.022**	**1.881**	**1.095–3.232**	**0.020**	**1.938**	**1.108–3.389**
BMI (median, IQR)	21.65 (18.80–24.20)	23.05 (19.90–25.80)	0.193	1.046	0.978–1.119			
History of previous abdominal surgery (count, %)	**59 (7.2)**	**14 (14.3)**	**0.017**	**2.141**	**1.146–3.999**	0.110	1.939	0.861–4.364
Previous episodes (count, %)	70 (8.6)	7 (7.1)	0.632	0.821	0.366–1.839			
ABT (past 30 days) (count, %)	26 (3.2)	5 (5.1)	0.326	1.636	0.613–4.362			
Duration of symptoms (count, %)								
< 24 h	**378 (46.3)**	**24 (24.5)**	**0.001**			**0.015**		
24–48 h	**190 (23.3)**	**30 (30.6)**	**0.002**	**2.487**	**1.414–4.373**	**0.013**	**2.261**	**1.191–4.294**
48–72 h	**110 (13.5)**	**17 (17.3)**	**0.008**	**2.434**	**1.262–4.693**	**0.043**	**2.181**	**1.025–4.642**
> 72 h	**139 (17)**	**27 (27.6)**	**< 0.001**	**3.059**	**1.707–5.482**	**0.003**	**2.873**	**1.436–5.751**
Fever (*T* > 37.5 °C)	161 (19.7)	27 (27.6)	0.071	1.549	0.963–2.493			
Mc Burney (count, %)	**517 (63.6)**	**50 (51)**	**0.016**	**1.677**	**1.101–2.555**	0.160	1.422	0.870–2.326
Blumberg (count, %)	355 (43.6)	34 (34.7)	0.095	1.453	0.937–2.252			
White blood cells (median, IQR)	14.47 (11.46–17.35)	14.57 (11.70–17.59)	0.388	1.020	0.976–1.066			
Neutrophils (median, IQR)	11.73 (8.62–14.54)	11.74 (9.22–13.97)	0.573	1.014	0.967–1.063			
CRP (median, IQR)	**3.03 (0.56–8.36)**	**6.13 (2.93–16.91)**	**< 0.001**	**1.055**	**1.034–1.077**	0.619	1.004	0.988–1.020
Alvarado risk class (count, %)								
Low risk	347 (42.5)	34 (34.7)	0.339					
Medium risk	374 (45.8)	51 (52)	0.157	1.392	0.880–2.200			
High risk	96 (11.8)	13 (13.3)	0.350	1.382	0.702–2.722			
Time delay to surgery (hours, median, IQR)	19.43 (11.17–30.18)	19.42 (10.50–29.59)	0.365	1.004	0.995–1.014			
Time delay to surgery (count, %)								
< 12 h	219 (26.8)	26 (26.5)	0.991					
12–24 h	278 (34)	34 (34.7)	0.914	1.030	0.600–1.769			
> 24 h	320 (39.2)	77 (38.8)	0.999	1.000	0.590–1.695			
Type of surgery (VLS, count, %)	692 (84.7)	77 (78.6)	0.120	0.662	0.394–1.113			
Open conversion (count, %)	**52 (7.5)**	**18 (23.4)**	**< 0.001**	**3.755**	**2.063–6.833**	0.342	1.579	0.615–4.054
Drainages (count, %)	**308 (37.7)**	**69 (71.1)**	**< 0.001**	**4.072**	**2.567–6.460**	**0.013**	**2.209**	**1.184–4.120**
Duration of surgery (min, median, IQR)	**70 (55–90)**	**85 (65–120)**	**< 0.001**	**1.016**	**1.011–1.022**	0.281	1.005	0.996–1.014
Complicated appendicitis (count, %)	**227 (27.8)**	**51 (52)**	**< 0.001**	**2.820**	**1.844–4.313**	**0.045**	**1.808**	**1.012–3.228**

Statistical significance (*p* < 0.05) is highlighted in bold

In the multivariate analysis, the preoperative variables that associated with postoperative complications were male sex [OR: 1.938 (CI 95% 1.108–3.389) *p* = 0.020] and duration of symptoms [24–48 h OR: 2.261 (CI 95% 1.191–4.294) *p* = 0.013; 48–72 h OR: 2.181 (CI 95% 1.025–4.642) *p* = 0.043; > 72 h OR: 2.873 (CI 95% 1.436–5.751) *p* = 0.003]. Among intraoperative variables, presence of drainage [OR: 2.209 (CI 95% 1.184–4.120) *p* = 0.013] and complicated appendicitis [OR: 1.808 (CI 95% 1.012–3.228) *p* = 0.045] were significantly related to postoperative complication at multivariate analysis.

The in-hospital surgery delay, examined both as a continuous and categorical variable, showed no association with postoperative morbidity. This lack of association remained consistent across all Alvarado risk class groups. In particular, timing considered as a continuous variable was not associated with postoperative morbidity in the low-, medium-, and high-risk classes, with *p* values of 0.78, 0.28, and 0.24, respectively. Moreover, when considering time as a categorical variable, for the low-risk group, the OR for surgery within 12–24 h was 0.2 (95% CI: 0.03–1.61), and for > 24 h, it was 1.56 (95% CI: 0.66–3.68). In the medium-risk group, the OR for surgery within 12–24 h was 1.39 (95% CI: 0.62–3.1), and for > 24 h, it was 1 (95% CI: 0.45–2.2). For the high-risk group, the OR for surgery within 12–24 h was 0.4 (95% CI: 0.1–1.67), and for > 24 h, it was 0.16 (95% CI: 0.02–1.54).

## Discussion

The present study indicates that in-hospital delay is not associated with the severity of appendicitis or the presence of postoperative complications, even across Alvarado risk class groups. Patients within the same Alvarado risk class group presented to the emergency room with similar clinical and radiological characteristics, thereby representing a more homogeneous population.

Predictors of complicated appendicitis include increasing age, duration of symptoms, and neutrophil absolute count, while predictors of morbidity consist of male sex and duration of symptoms.

The optimal timing of appendicectomy for acute appendicitis is a controversial issue. A systematic review by van Dijk et al. [2] has shown that delaying appendicectomy for up to 24 h after admission is not linked to a higher risk of complicated appendicitis, postoperative surgical-site infection, or morbidity. In another study, Alore et al. [3] analyzed the American College of Surgeons National Surgical Quality Improvement Program database for appendectomies performed between 2012 and 2015. In this period, over 112,000 patients underwent non-elective appendectomy for acute appendicitis. They found that only appendicectomies performed 48 h after admission were associated with a higher risk of major postoperative complications (8%) in comparison with appendicectomies performed within 24 h and 48 h after admission (3.4% and 3.6%, respectively).

In contrast, several studies have found that delayed appendicectomies are associated with poor outcomes. A multicenter study [4] analyzed 1,675 patients who underwent appendicectomy in 11 Swiss hospitals between 2003 and 2006. They found that appendicectomies performed 12 h after admission were associated with a higher risk of perforated appendicitis (29.7% vs. 22.7%). Moreover, a multicenter study [5] has shown that perforation rate was 28.8% for appendicectomies performed within 24 h and 33.3% for those performed between 24 and 48 h with an OR of 1.2 for adults and 1.08 for children.

In our experience, in-hospital delay was not a determinant of complicated appendicitis nor with postoperative complications. Even when patients were stratified on preoperative risk of acute appendicitis (Alvarado score classes), surgical delay was not associated with the outcome. Therefore, our study contributes additional evidence supporting the notion of delaying appendicectomy for up to 24 h.

Our results are consistent with those of Maru Kim et al. [11] who found no significant association between complicated appendicitis and in-hospital delay (398.7 ± 154 min vs. 402 ± 194.9 min). Moreover, they found no significant association between complicated appendicitis and in-hospital delay considered as a categorical variable with a cut-off of 6 h (RR 0.83 CI 95% 0.62–1.13) and 12 h (RR 1.5 CI 95% 0.66–3.38). In addition, Abou-Nukta et al. [12] found no significant association between appendicectomies performed between 12–24 h after admission and postoperative complications such as wound infection and abscess formation (*p* value 0.74).

Then we examined the possible predictive factors for complicated appendicitis. Increasing age, duration of symptoms, and neutrophil level were statistically associated with the severity of appendicitis at multivariate analysis. It is intriguing to note that the duration of symptoms was associated with the severity of appendicitis, whereas in-hospital delay did not show significant correlation. This implies a need to redirect research attention from intra-hospital delay, which has been extensively addressed in the studies mentioned previously [2–5, 11, 12], to considering the impact of extra-hospital delay. This aspect of research could be explored in another future study.

These results were similar to those of Fujiwara et al. [13] who examined different predictive factors for complicated appendicitis. The significant factors included aged > 40 years (66.7% vs 41.1, *p* value = 0.002) and duration of symptoms > 24 h (67.3% vs 29.8%, *p* value < 0.001).

Preoperative variables associated with postoperative complications were male sex and duration of symptoms at multivariate analysis. The time from the onset of symptoms and in-hospital presentation were significantly related with postoperative complication occurrence.

Among intraoperative factors, only the usage of drainages and the presence of complicated appendicitis were associated with an increased risk of develop a postoperative complication.

The present study has some limitations. This is a retrospective analysis of a large prospective register and it cannot be excluded that there is a presence of a selection bias. However, data were confirmed even when patients were stratified by preoperative risk of severity.

## Conclusion

Based on these findings, it appears to be reasonably safe to delay appendicectomy for acute appendicitis for up to 24 h from hospital admission. In other words, surgeons could safely postpone appendicectomy to the next morning if a patient shows up with acute appendicitis during night shifts. Furthermore, our results suggest that the timing for appendicectomy in acute appendicitis should be calculated from symptoms onset rather than hospital presentation.

In conclusion, the present study showed no association between in-hospital delay and the severity of appendicitis or the occurrence of postoperative complications. Factors such as advancing age, a longer duration of symptoms, and a higher neutrophil count were identified as predictors of complicated appendicitis. In addition, male gender and a prolonged duration of symptoms were identified as predictors of morbidity.

## Data Availability

Data can be obtained under request to the corresponding author.
